# Deep Learning Analyses to Delineate the Molecular Remodeling Process after Myocardial Infarction

**DOI:** 10.3390/cells10123268

**Published:** 2021-11-23

**Authors:** Oriol Iborra-Egea, Carolina Gálvez-Montón, Cristina Prat-Vidal, Santiago Roura, Carolina Soler-Botija, Elena Revuelta-López, Gemma Ferrer-Curriu, Cristina Segú-Vergés, Araceli Mellado-Bergillos, Pol Gomez-Puchades, Paloma Gastelurrutia, Antoni Bayes-Genis

**Affiliations:** 1 ICREC Research Program, Health Sciences Research Institute Germans Trias i Pujol, Universitat Autònoma de Barcelona, 08916 Barcelona, Spain; oiborra@igtp.cat (O.I.-E.); cgalvez@igtp.cat (C.G.-M.); cpratv@gmail.com (C.P.-V.); sroura@igtp.cat (S.R.); csoler@igtp.cat (C.S.-B.); erevuelta@igtp.cat (E.R.-L.); gferrer@igtp.cat (G.F.-C.); mamellado@igtp.cat (A.M.-B.); pgomez@igtp.cat (P.G.-P.); pgastelurrutia@igtp.cat (P.G.); 2 CIBERCV, Instituto de Salud Carlos III, 28029 Madrid, Spain; 3 Faculty of Medicine, University of Vic-Central University of Catalonia, 08500 Vic, Spain; 4 Anaxomics Biotech S.L., Diputació 237 1r 1a, 08007 Barcelona, Spain; cristina.segu@anaxomics.com; 5 Institut d’Investigació Biomèdica de Bellvitge-IDIBELL, 08007 Barcelona, Spain; 6 Cardiology Service, Germans Trias i Pujol University Hospital, 08916 Badalona, Spain; 7 Department of Medicine, Autonomous University of Barcelona, 08916 Barcelona, Spain

**Keywords:** myocardial infarction, deep learning, gene regulation, transcriptomics

## Abstract

Specific proteins and processes have been identified in post-myocardial infarction (MI) pathological remodeling, but a comprehensive understanding of the complete molecular evolution is lacking. We generated microarray data from swine heart biopsies at baseline and 6, 30, and 45 days after infarction to feed machine-learning algorithms. We cross-validated the results using available clinical and experimental information. MI progression was accompanied by the regulation of adipogenesis, fatty acid metabolism, and epithelial–mesenchymal transition. The infarct core region was enriched in processes related to muscle contraction and membrane depolarization. Angiogenesis was among the first morphogenic responses detected as being sustained over time, but other processes suggesting post-ischemic recapitulation of embryogenic processes were also observed. Finally, protein-triggering analysis established the key genes mediating each process at each time point, as well as the complete adverse remodeling response. We modeled the behaviors of these genes, generating a description of the integrative mechanism of action for MI progression. This mechanistic analysis overlapped at different time points; the common pathways between the source proteins and cardiac remodeling involved IGF1R, RAF1, KPCA, JUN, and PTN11 as modulators. Thus, our data delineate a structured and comprehensive picture of the molecular remodeling process, identify new potential biomarkers or therapeutic targets, and establish therapeutic windows during disease progression.

## 1. Introduction

Myocardial infarction (MI) occurs when blood flow suddenly stops due to occlusion of a coronary artery, leading to local ischemia in the heart [1,2,3]. This condition triggers an adverse myocardial remodeling response involving a wide variety of signaling pathways, potentially including extracellular matrix (ECM) dysregulation, cardiomyocyte apoptosis, and cardiogenic processes, such as myocyte concentric and eccentric hypertrophy, slippage, accumulation of interstitial tissue, a molecular shift towards an embryonic pattern, and loss of cardiac energy reserves [4,5]. Cardiac remodeling secondary to MI is a well-defined process that may ultimately lead to heart failure (HF) [6,7,8], but the specific dynamic molecular mechanisms underlying this progression have not been fully characterized. This process has been examined in several studies using small experimental animal models, mainly providing data about individual genes or proteins [9,10,11,12]. Although these investigations have provided valuable information regarding cardiac remodeling, they have been insufficient for capturing this complex process as a whole.

Increasing evidence supports the idea that specific biological processes (e.g., protein–protein interactions or epigenetic regulation) are likely influenced by the biological context, such as a specific tissue or a certain disease [13,14,15,16]. Compared to small animal models, large experimental animal models more closely resemble human cardiac physiology, function, and anatomy; therefore, their use constitutes a key step in translating experimentally obtained information into clinical use. In addition, vast amounts of data are constantly being generated, and compiling, analyzing, and interpreting this information as a whole constitutes an overwhelming task. For this purpose, new technologies are rapidly emerging that combine different engineering approaches and bioinformatics. Within this context, systems biology arose as an interdisciplinary field of study with the aim of unravelling the key interactions within complex biological networks following a holistic approach based on computational and mathematical models.

**Figure 1:**
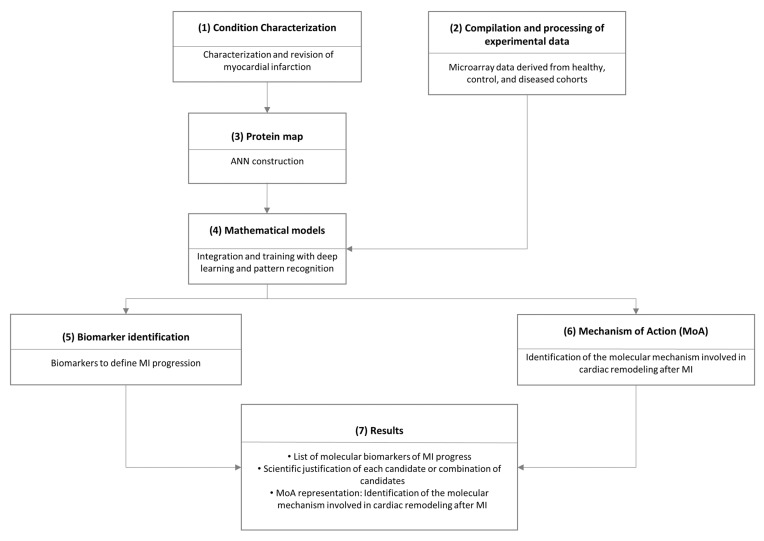
Schematic representation of the implemented systems biology workflow. (1) First, we characterized myocardial infarction (MI) at the molecular level via manual curation of the literature and using a compendium of massive public databases describing the molecular interactions of interest. (2) In parallel, we used experimental transcriptomic data to define the molecular behavior of MI at each time point. (3) We used the information to generate a map of proteins regulated by the obtained experimental data. (4) We fed the mathematical models to identify patterns in the data, (5) identify key regulatory proteins, and (6) predict new mechanisms of action. (7) The final result included a scientific justification of every prediction. ANN: artificial neural network.

Using transcriptomic information from infarcted and remote in vivo swine heart tissues, we extensively analyzed the temporal and region-specific myocardial gene expression patterns in response to MI [17]. To determine the underlying molecular cause of the observed cardiac remodeling, we incorporated this transcriptomic data into a deep learning model, the Therapeutic Performance Mapping System (TPMS^®^) (Figure 1) [18]. TPMS^®^ applies artificial intelligence and pattern recognition techniques to combine interactomic data with the molecular and clinical responses observed in patients. First, the interactomic information is used to generate a skeleton for computational models, which act as a network of potential mechanistic interactions. Second, this network is fitted into a deep learning model and trained using clinical and molecular responses observed in patients. This ultimately results in the generation of a mathematical model capable of both reproducing existing knowledge and discerning the mechanisms of action hidden under thousands of molecular interactions that are otherwise inaccessible.

In the present study, we incorporated transcriptomic data obtained from infarcted porcine hearts at different time points into their corresponding mathematical configurations to gain insights into the molecular evolution of MI over time and its development into HF.

## 2. Materials and Methods

### 2.1. Myocardial Infarction Model

Female Landrace × Large White pigs (*n* = 9) were premedicated with intramuscular (IM) azaperone (10 mg/kg) (Stresnil^®^, Laboratorios Esteve, Barcelona, Spain), and then administered intravenous (IV) pentobarbital sodium (15 mg/kg) (Tiobarbital^®^ 1 g, B. Braun, Melsungen, Germany). These pigs underwent endotracheal intubation with 2% inhaled isoflurane as the anesthesia. During the procedure, IV fentanyl (0.75 mg/kg/45 min) (Fentanest^®^, Kern Pharma, Madrid, Spain) was administered as an analgesic, and IV atracurium besylate (1.5 mg/kg bolus) (Tracrium^®^, GlaxoSmithKline, Brentford, UK) was used to induce muscular relaxation. After a left lateral thoracotomy, MI was induced via permanent ligation of the circumflex artery as described previously [19]. IM tulathromycin (2.5 mg/kg) (Draxxin^®^, Pfizer Animal Health, New York, NY, USA) was administered as an antibiotic prophylactic, and a transdermal fentanyl patch was applied to facilitate post-operative analgesic care. These surgical interventions were monitored by electrocardiogram (ECG), capnography, and pulse oximetry, and with noninvasive measurement of arterial blood pressure and temperature.

Six days (*n* = 3), 30 days (*n* = 3), or 45 days (*n* = 3) after MI, animals were randomly sacrificed by an IV overdose of potassium chloride solution. At each temporal stage, we analyzed three paired myocardial samples from the infarct core and non-infarcted remote myocardium. As a physiological control, we analyzed myocardial samples from healthy animals (*n* = 3).

### 2.2. Tissue Collection and RNA Extraction

Following sternotomy, hearts were washed in ice-cold buffered saline solution to remove blood residue. Biopsies were obtained from the infarct core (center of the scar on the left ventricle), remote myocardium (non-infarcted interventricular septum), and control myocardium (healthy animals). To ensure RNA stabilization, biopsies were preserved in Allprotect Tissue Reagent (Qiagen, Hilden, Germany) at room temperature. Total RNA was isolated using the RNeasy Fibrous Tissue Mini Kit (Qiagen, Hilden, Germany). RNA purity and integrity were assessed by spectrophotometry (NanoDrop ND-1000, NanoDrop Technologies, Thermo Fisher Scientific, Waltham, MA, USA) and nanoelectrophoresis (2100 Bioanalyzer, Agilent Technologies, Santa Clara, CA, USA).

### 2.3. Microarray Gene Expression Analysis

We obtained microarray expression profiles using the GeneChip^®^ Porcine Genome Array (Affymetrix). We processed 200 ng of total RNA from each sample, labeling, fragmenting, and hybridizing it to the GeneChip^®^ following the manufacturer’s instructions. Arrays were scanned using an Affymetrix GeneChip^®^ Scanner 3000 7 G. The raw expression data were preprocessed using the robust multichip average (RMA) normalization method [20], yielding 24,123 probe sets on a log2 basis.

### 2.4. Compilation of Transcriptomic Data

First, the data were filtered to discard all entries with contradictory information (i.e., two entries for the same gene name with negative and positive ratio values) and to identify the number of uniquely altered genes. Next, the swine transcriptomics were translated into their human equivalents via reciprocal best hits (RBHs) with BLAST and Gene Name Correspondence. Pig-to-human RBHs were identified using the InParanoid database [21]. We only included genes with an associated protein product. Gene information was mapped one-to-one to its protein product for its introduction into the protein network. For each gene, the correlation between RNA and protein levels was assessed using an RNA-to-protein ratio, as described previously [22]. If RBHs were not found for a protein, we used the reviewed UniProt entry for the human protein with a matching gene name (Supplementary Table S1). Next, the proteins were labeled according to whether a protein is activated or inhibited under physiological conditions, and we used this information as a reference for restricting detection to the variability from these values, as defined by *p*-values adjusted using the Benjamini–Hochberg procedure to control the false discovery rate (FDR) at 0.01 [23,24]. Finally, proteins with human UniProt IDs within each cohort were used as molecular restrictions for our models.

### 2.5. Molecular Characterization of Pathology

We integrated published data with our results to define a set of molecular profiles characterizing MI and adverse myocardial remodeling, which were used to build the protein network and mathematical model.

We carefully and extensively reviewed articles in the PubMed database (abstract or full-text depending on the inclusion of information on the molecular definition of disease) that included the following search strings: myocardial infarction—“myocardial infarction”[title] AND (“pathology”[All Fields] OR “physiology”[All Fields] OR “pathophysiology”[All Fields]) AND “molecular”[All Fields] AND (Review[ptyp] AND English[lang]); cardiac remodeling—(“cardiac remodeling”[title] OR “cardiac remodelling”[title]) AND (“pathology”[All Fields] OR “physiology”[All Fields] OR “pathophysiology”[All Fields]) AND “molecular”[All Fields] AND English[lang]). If the evidence for candidate proteins was not enough to include them on the effector list, specific searches for the protein were performed to assess its validity. We further characterized the pathophysiological processes at the protein level using a set of 202 unique proteins (121 related to MI and 136 to cardiac remodeling, with some overlap) that centered our analysis on the pathological conditions of interest in the human biological network.

### 2.6. Therapeutic Performance Mapping System (TPMS) Generation of Mathematical Models

We studied the pathology at 6, 30, and 45 days of evolution in samples from the infarct core (C6, C30, and C45, respectively), which included 4737, 4730, and 4203 differentially expressed proteins, respectively. We also studied samples from the remote area surrounding the infarct localization at the same time points (R6, R30, and R45, respectively), which included 122, 117, and 21 differentially expressed proteins, respectively. Using these data, we identified the main pathophysiological processes that were altered in MI and cardiac remodeling.

We trained the mathematical models using a large collection of well-established pathophysiological signals and clinical information relevant to the examined pathologies. Molecular descriptions of these input-output signals were obtained primarily via manual literature mining and from a compendium of massive databases that accumulate biological and clinical knowledge (e.g., KEGG, MINT, REACTOME, BIOGRID, and DrugBank). The model inputs included information on drugs, pathologies, and protein/gene relationships that could inhibit or activate one or more nodes of the protein network (their targets), triggering a perturbation through the system. The model outputs were experimental microarray data regarding upregulated or downregulated genes/proteins and clinical information. Using the model’s input and output data, mathematical algorithms were generated to trace a change or perturbation from one to another, elucidating the mechanism of action explaining this connection.

The generated collection of known input-output physiological signals can be envisioned as a list of physiological principles that are representative of all humans or of particular pathophysiological conditions. These sets of rules are collated to form a “truth table” that every constructed mathematical model must satisfy. Transcriptomic information was compiled, analyzed, and included in the models when it met the reliability requirements described above.

The constructed models were used to determine the weight of the relative value of each protein (node) relationship. However, the very high number of links exponentially increased the number of parameters to solve. To optimize the system, we used two different approaches: one based on randomized systems (Monte Carlo-based system) [25], and the other based on information derived from the network topology [26].

### 2.7. Analyzing and Solving the Mathematical Models

The TPMS technology employs two different but complementary strategies to solve mathematical models. The artificial neural network (ANN) strategy can identify relationships among regions of the network (generalization). This strategy provides a predictive value that infers the probability that a specific relationship exists between two or more sets of proteins. In this case, we tested each differential protein against the described cardiac remodeling signature. Next, the model’s predictive capacity is cross-validated using different sets of data towards what is described in the literature and databases.

The second strategy is the sampling method, which allows the observed effects to be traced back to specific molecules or drugs. Sampling methods are only applied once a key region of the protein map has been identified using ANNs or is suggested by experimental work. Once a response (indication, adverse effects, specific clinical improvements, etc.) has been identified and linked to a specific stimulus (compound, pathology, protein expression alteration, etc.) using ANNs, sampling methods enable analysis of the mechanism of action and elucidation of the hidden relationships between them.

### 2.8. Biomarker Identification

Using the characterization of MI, we performed multivariate analysis to evaluate combinations of two, three, or five proteins and identify the combinations that best classified either the solutions of the models or the microarray experiments. To assess the probability of correctly predicting and classifying the set of samples of high-throughput data, we used a “leave one out” strategy, which involved analyzing a subset of samples that were not previously included in the models.

The combinations were obtained via four strategies. To classify the solutions of the models, we used a model-based strategy and a model/HT-based strategy. Both strategies identified the protein combinations that better classified the solutions of the models to their corresponding cohort. The model/HT-based strategy additionally filtered the protein combinations using the high-throughput data (i.e., what was measured in the microarray), reducing the number of proteins and facilitating the identification of a combination with a higher generalization capability.

To classify the microarray experiments, we used an HT-based strategy and an HT/model-based strategy. Both strategies identified the combinations of proteins that better classified the microarray experiments to their corresponding cohort. The HT-based strategy did not use the information from the disease models, whereas the HT/model-based strategy filtered the combinations by the proteins found to be relevant in the models.

Using these strategies, we generated a list of 106 individual proteins that represent potential biomarkers of the description of the pathology at a given time point (Supplementary Table S2).

### 2.9. Generating A Molecular Model of Cardiac Remodeling

Starting from the differential proteins for each cohort, we performed a relationship analysis with ANNs to identify proteins mechanistically related to cardiac remodeling. Proteins having a predictive value > 50% (corresponding to ~20% probability of accepting false positives) were included for further evaluation (Table 1). Upon identifying the differential proteins more closely related to cardiac remodeling, two analyses were performed. A gene set enrichment analysis (GSEA) based on functional annotation of the differentially expressed genes was used to identify relevant biological processes (KEGG pathways, Gene Ontology (GO) function, and GO process terms; Supplementary Table S3) [27,28]. We also applied triggering-protein analysis to identify the key proteins in a network that strongly affect the rest of the dataset (i.e., the proteins triggering the observed output). These proteins were further investigated and sampling methods used to explore their exact relationship to cardiac remodeling.

### 2.10. Data Integration

We integrated the in silico analysis of the microarray gene expression data to identify relationships across signature datasets, providing an independent assessment and functional validation. To cross-validate our findings, we used several complementary methods for complex data integration. The Metascape bioinformatics tool [29] was used for pathway analyses, the Perseus software platform (V1.6.5.0) [30] for high-dimensional omics data analysis, and the STRING [31] online tool to explore relevant protein–protein interactions (PPI) at 0.9 confidence.

## 3. Results

### 3.1. High-Dimensional Data Integration of Microarray Gene Expression during 6 Weeks of MI Progression

Each distinct tissue dataset (C6, C30, C45, R6, R30, and R45) revealed substantial changes over 6 weeks of MI progression. To determine whether these changes were consistent across data types and to validate their time-dependent evolution in functional pathways, we adopted three independent strategies.

**Figure 2:**
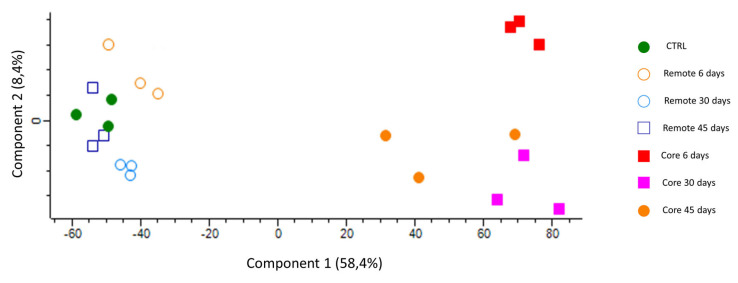
Principal component analysis. The visual output showing perfect separation of the six groups and the two different regions (upper left plot).

First, we sought to assess the internal structure of the data in a way that best explained its variance. Due to the high complexity of the datasets, we used the Perseus platform to perform principal component analysis (PCA) for the reduction of dimensionality. This approach clearly separated the core infarcted area from the remote area and the control group, which clustered together (Figure 2).

**Figure 3:**
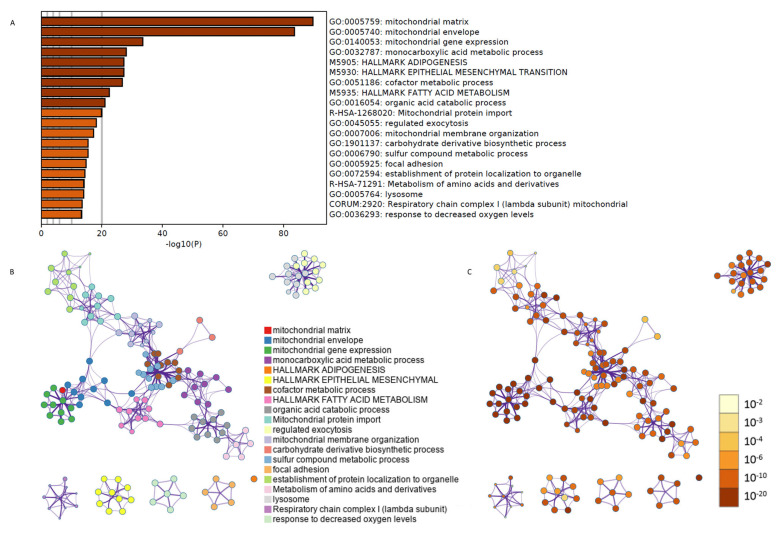
Functional validation of the integration of gene expression data over 6 weeks after myocardial infarction. Using Metascape algorithms, we identified the most affected biological processes according to their GO terminology (**A**). These processes were then clearly clustered using a network analysis based on their biological significance (**B**) and *p*-values (**C**).

Second, we employed Metascape algorithms to sort out the most affected pathways in the gene set according to their GO terms (Figure 3A). We were able to further cluster them according to their biological significance and associated *p*-values (Figure 3B,C). These data suggested that MI progression was accompanied by a great alteration in mitochondrial metabolism, as well as in the regulation of adipogenesis, fatty acid metabolism, and epithelial–mesenchymal transition processes.

**Figure 4:**
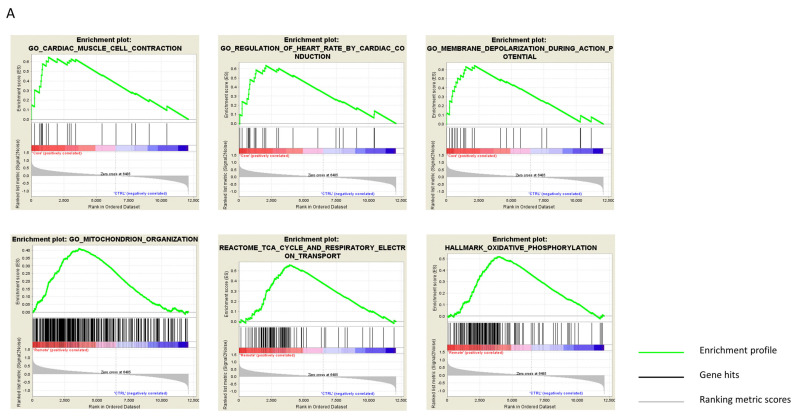

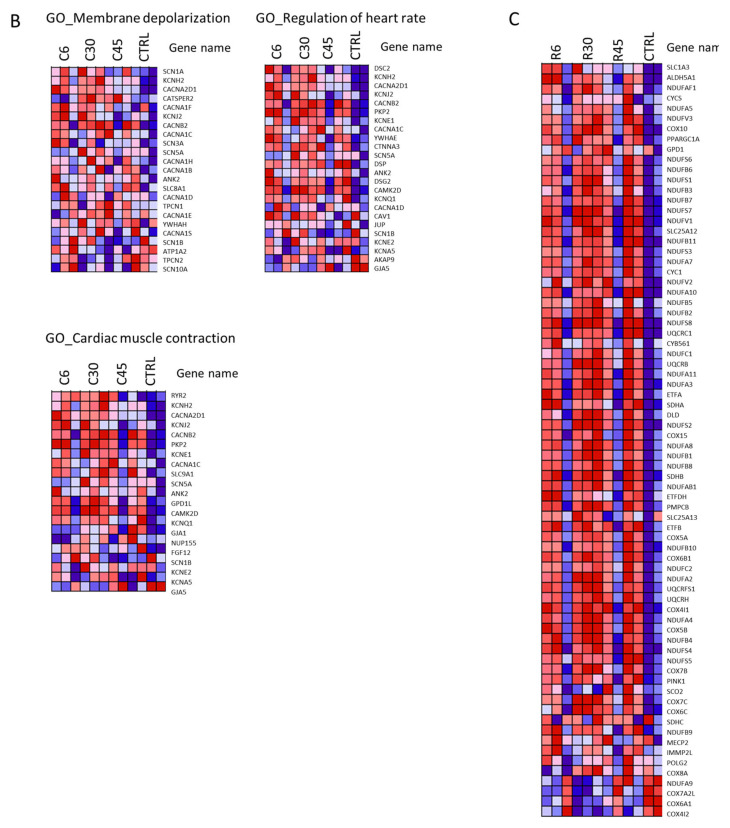
Enrichment and overlap analyses and heatmap clustering. (**A**) Results of the enrichment and overlap analyses. Upper panels: infarct core region. Lower panels: remote myocardium region. The enrichment score (ES) reflects the degree to which a gene set is over-represented at the top or bottom of a ranked list of genes, with a positive or negative ES indicating gene set enrichment at the top or bottom of the list, respectively. ES is calculated by walking down the ranked list of genes, increasing a running-sum statistic when a gene is in the gene set and decreasing it when it is absent. The magnitude of the increment depends on the correlation of the gene with the phenotype. The ES is the maximum deviation from zero encountered in walking the list. (**B**,**C**) Heatmap clustering. (**B**) The clustered genes in the leading-edge subsets in the upper panels in (**A**) (core region). (**C**) The clustered genes in the leading-edge subsets in the lower panels in (**a**) (remote region). Expression values are represented by colors, with red, pink, light blue, and dark blue indicating high, moderate, low, and lowest expression, respectively.

Finally, GSEA and overlapping tests allowed the contextualization of this information and distribution of the GO terms across core vs. control, and remote vs. control datasets. This revealed intrinsic differences between the core and remote regions of the heart after MI (Figure 4A–C). Specifically, the core region exhibited greater deterioration of cardiac contraction, depolarization during action potentials, and of heart rate regulation (Figure 4A, upper panels), whereas the remote region exhibited a heavy enrichment of cellular respiration processes (Figure 4A, lower panels).

### 3.2. Time-Dependent Identification of MI-Derived Biomarkers Strongly Related to Cardiac Remodeling

**Figure 5:**
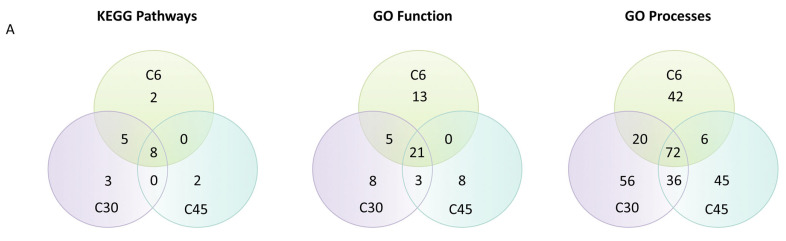

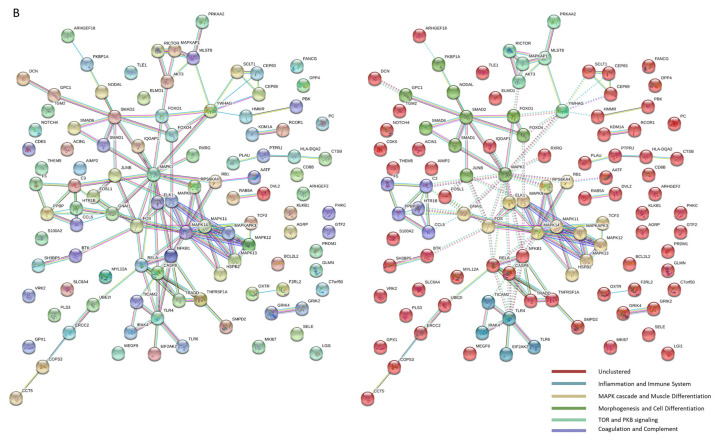
Protein–protein interaction network analysis. (**A**) Venn diagram depicting the common enriched pathways between the three different time points. (**B**) Network analysis using STRING software, including the 105 protein candidates identified by artificial intelligence analysis techniques. Left: the cloud of interactions at 0.9 evidence. Right: the same interactions clustered by *k*-means for vector quantization at *K* = 6. Number of nodes: 105; number of edges: 128; average node degree: 2.44; average local clustering coefficient: 0.469; expected number of edges: 62; PPI enrichment *p*-value: 1.32 × 10^−7^.

To specifically investigate the adverse cardiac remodeling processes that occur after MI, we integrated the high-throughput microarray data with molecular information available in the literature. Analysis of all tissues (infarcted core and remote) and time points (6, 30, and 45 days) identified 105 proteins (Figure 5B; Supplementary Table S2). Of these, 42 (40%) were previously described in the literature as being related to MI and showed associations with adverse remodeling (DPP4, TNR1A, and IRAK4), apoptosis and survival (IQGA1, RICTR, p38, JNK, FADD, FOXO1, CASP8, ACINU, and CSN3), inflammation and anti-inflammatory processes (LYAM2, TCAM2, AKT3, CCL5, C3, NFKB1, TF65, TNR1A, CXCL7, IRAK4, FOXO4, TLR4, S10A2, and LST8), fibrosis (ERK, PGS2, SMAD6, SMAD2, and LST8), impaired contractility (ML12A), regeneration and cell proliferation (HMMR, KI67, SMAD1, and CSN3), heart development (SMAD6 and PRDM1), metabolism shift (AAPK2), and cardiac hypertrophy (CATB, IQGA1, and CSN3).

Notably, seven proteins overlapped between the different time points regardless of the myocardial region (Table 2). Of the 105 proteins, 52 (49.5%) were recognized by both the models and the transcriptomic data analysis (model/HT or HT/model identification method). As these proteins were identified in models of both the pathology and the experimental data, they were considered more robust. Moreover, we found two distinct combinations of proteins from the infarct core data at each time point, which had generalization capabilities and accuracies of 100% (*p* ≤ 0.05; Table 3). No reliable combinations were found using remote myocardium data.

### 3.3. Description of the Molecular Mechanistic Relationships Defining MI Evolution

Using the differentially expressed proteins found to be related to cardiac remodeling in the ANN analysis, we performed enrichment analysis to identify and understand the mechanism of action of cardiac remodeling in the infarct core at each time point. We identified a total of 355 processes that were altered in the infarct core region for at least one time point (221 upregulated and 134 downregulated). As shown in Figure 5A and Supplementary Table S3, there was a substantial overlap between the processes altered in the infarct core region at all three time points (C6, C30, and C45), including 8 KEGG pathways, 21 GO function terms, and 72 GO process terms. Other processes were specifically altered at one time point or two time points—mainly during the C6–C30 and C30–C45 transitions, although six GO process terms overlapped between C6 and C45.

Importantly, we found that 101 processes were altered in the infarct core throughout the progression to 45 days postinfarction. Most of the upregulated processes were related to ECM formation (organization, assembly, and adhesion) and to ECM components (hyaluronic, heparin, collagen, and glycosaminoglycan-related processes). We also detected alterations in processes related to muscle contraction, with an upregulation of actin- and calcium-related processes and downregulation of cardiac muscle contraction. Most of the downregulated processes were related to metabolism.

**Figure 6:**
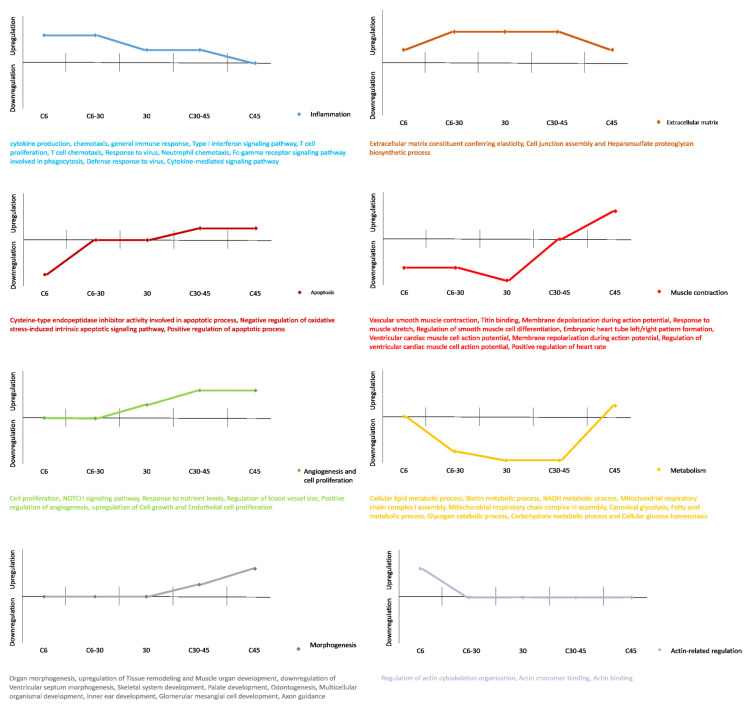
Graphic representation of the evolution of affected processes throughout the progression of myocardial infarction. Values on the X-axis show time evolution. Values on the Y-axis indicate upregulation (*v* > 0), downregulation (*v* < 0), or a lack of differential expression (*v* = 0 and matching the X-axis).

Some of the altered processes exhibited time-dependent behavior. Inflammation clearly appeared to be upregulated 6 days post-MI and decreased in later stages. Processes related to angiogenesis, cell proliferation, and morphogenesis started to increase 30 days post-MI and become more relevant by 45 days post-MI. Apoptosis also appeared to gain importance in the late stages. The upregulated pathways at 45 days suggested some normalization of metabolism, though several processes remained altered (Figure 6).

### 3.4. Identification of Key Proteins Driving Post-MI Alterations

After the mechanistically affected pathways were identified, triggering-protein analysis allowed us to determine the mechanistic relationship between paired sets of proteins. Each analyzed time point presented a list of particular source proteins (Table 4) with some degree of overlap between the different cohorts. Overall, the transcriptomic experiments in the infarct core region identified 20 proteins (primarily involved in growth factor signaling) as the main drivers, or source proteins, of post-MI alterations. KPCA, PTN11, JAK2, 1433B, ERBB2, VEGFA, JUN, RAF1, and IGF1R were identified as source proteins at all three time points. MAPK03, SRC, STAT1, NGF, RAC1, and TF65 were identified as common source proteins in C6 and C30. CBLB was common to C6 and C45, though its activity changed from day 6 (activated) to day 45 (inhibited). GCR, CDC42, and GRB2 were identified as source proteins only in C30, whereas MAPK01 was found specifically in C45.

### 3.5. Unraveling the Molecular Mechanism of Action Relating the Source Proteins to Cardiac Remodeling

**Figure 7:**
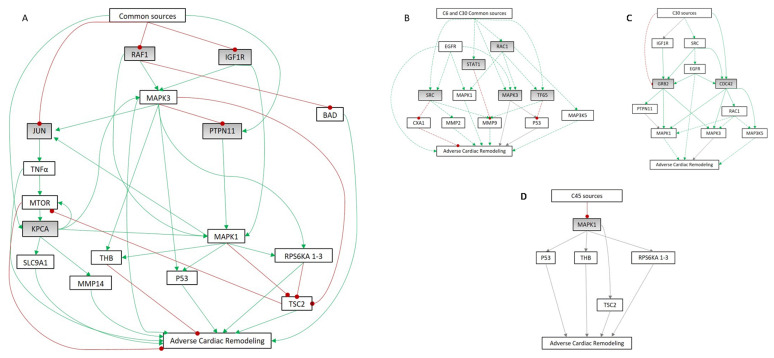
Mechanisms of action determined by artificial neural network (ANN) analysis. (**A**) Common mechanistic relationship between cardiac remodeling and the common source proteins (in grey: IGF1R, RAF1, KPCA, JUN, and PTN11) identified at all three analyzed time points in the infarct core region. (**B–D**) Mechanistic representation of (**B**) C6–C30 common source proteins (SRC, STAT1, MK03, RAC1, and TF65), (**C**) C30 source proteins (GRB2 and CDC42), and (**D**) C45 source proteins (MK01). Continuous colored lines depict links present only in one cohort. Discontinued lines depict links present in two cohorts. Continuous grey lines depict links present at all time points. C6: infarct core area 6 days after infarction; C30: infarct core area 30 days after infarction; C45: infarct core area 45 days after infarction.

Mechanistic analysis of the relationship between the identified source proteins and cardiac remodeling revealed an overlap between the different time points. The common pathways between the source proteins and cardiac remodeling (Figure 7) involved IGF1R, RAF1, KPCA, JUN, and PTN11. Analysis of the downstream signaling revealed a mechanism of action potentially involving 18 proteins, 15 of which were differentially expressed during at least one time point (Table 5). The ERK kinases MAPK01 and MAPK03 were apparently activated by PTPN11 and PKCA, respectively, and both were upregulated at all three time points. The other upstream proteins (IGF1R and RAF1) were inhibited. In addition, PTPN11 was functionally inhibited by MAPK03, resulting in a reduction in MAPK01 activity.

With regard to cellular proliferation, survival, and differentiation, we found that TSC2 was upregulated in C45. We also detected heavy upregulation of the ECM remodeling protein MMP14 and of BAD and P53, which are key components of apoptosis and survival. Accordingly, at all three time points, we detected downregulation of MTOR, SL9A1 (hypertrophy-related), and THB (impaired myocyte contractility).

## 4. Discussion

Our first goal was to assess the data structure of the transcriptomic information obtained in vivo. We integrated this highly dimensional data and analyzed its composition, revealing that MI progression was accompanied by a regulation of adipogenesis, fatty acid metabolism, and epithelial–mesenchymal transition processes. Moreover, this analysis uncovered robust segregation between the infarct core samples versus the remote myocardium and controls, indicating major differences between these areas at the regulatory level. We specifically investigated the biological significance underlying these structural variations, and found that the infarct core region of the heart was enriched in affected processes relating to heart muscle contraction and membrane depolarization. In contrast, the remote myocardium was heavily affected by respiratory metabolism deficiencies. These results helped contextualize the MI.

Adverse ventricular remodeling is a hallmark of MI evolution and progression towards HF [32,33,34]; therefore, we investigated which of the identified mechanisms were related to adverse cardiac remodeling. Using machine learning techniques to analyze all tissues and time points, we identified 105 altered genes that are most likely related to cardiac remodeling in MI. This provided a list of potential biomarkers that may indicate the pathological evolution according to each time point. Further analysis revealed two distinct combinations of genes from the infarct core data at each time point, that exhibited generalization capabilities and accuracies of 100%, suggesting that these genes are of great importance in describing the whole progression.

With this information, we performed enrichment analyses to examine the mechanism of action of cardiac remodeling in the infarct core at each time point. A total of 355 processes were altered in the infarct core for at least one time point, and 101 were altered at all time points (i.e., 6, 30, and 45 days after infarction). The high number of upregulated ECM-related processes was consistent with previous reports that glycosaminoglycan mediates cardiac remodeling by facilitating the inflammatory response [35]. In addition to its role in remodeling, the ECM also plays an important role in cell communication [36] and contributes to helping damaged cardiac tissue. To avoid ischemic conditions, the organism will attempt to revascularize and repair the affected area, promoting angiogenesis. Accordingly, angiogenesis was among the first morphogenic responses that we detected as being sustained over time. We also detected the apparent upregulation of other processes related to the morphogenesis of different organs, suggesting that postischemic cells present in the heart could be trying to recapitulate embryogenic processes and bolster regeneration.

Upon identification of the altered processes, we performed a protein-triggering analysis to establish the key genes mediating the adverse remodeling response. We then modeled the behaviors of these genes, generating the first description of an integrative mechanism of action for MI progression. This mechanistic analysis overlapped at different time points, and the pathways that were common between the source proteins and cardiac remodeling involved IGF1R, RAF1, KPCA, JUN, and PTN11 as modulators. The downstream signaling of these proteins produces a mechanism of action potentially involving 18 proteins, 15 of which were differentially expressed in at least one cohort.

The present study is the first to perform an in-depth analysis of the molecular changes characterizing the progression of MI. Our results contain a large amount of intricate information. As expected, MI evolution encompasses a myriad of altered processes and specific proteins that regulate time-dependent stages and determine the final extent of the pathology.

## 5. Conclusions

We have integrated dynamic transcriptomic regulation and all available information on MI and cardiac remodeling to delineate a clear, structured, and simplified picture of the complete post-MI remodeling process. Furthermore, our study also elucidates new potential targets and therapeutic windows for the treatment of MI complications and novel avenues for MI research, with the ultimate goal of fully unravelling the whole pathology.

## Figures and Tables

**Table 1:** Number of proteins mechanistically related to cardiac remodeling.

Protein Localization	C6	C30	C45	R6	R30	R45
# of proteins mechanistically related to cardiac remodeling	3302	3267	2930	83	77	13

C: infarct core area; R: remote area; 6: 6 days after infarction; 30: 30 days after infarction; 45: 45 days after infarction.

**Table 2:** Proteins that overlap between time points regardless of the myocardial region.

Protein Information		Identification Method	Identified as Classifier						Secreted	Related to MI
UniProt	Protein Name		C6	C30	C45	R6	R30	R45		
P27487	DPP4	Models, models/HT	1	1	1	−	1	1	1	1
Q6R327	RICTR	Models	1	1	1	−	−	1	0	1
Q15759	MK11	Models	1	1	1	−	−	−	1	1
P53778	MK12	Models, models/HT, HT/models	1	−	1	1	1	−	1	1
P07585	PGS2	Models	−	1	1	1	−	1	1	1
O75676	KS6A4	Models	−	1	1	1	−	−	1	0
Q9UKL0	RCOR1	Models	−	−	1	1	1	−	1	0

Models: the combinations of proteins that better classify the solution of the model to the corresponding cohort; models/HT: the combinations of proteins that better classify the solution of the model to the corresponding cohort filtered by the proteins acting according to the high-throughput (HT) data; HT/models: the combinations of proteins that better classify the microarray experiments to the corresponding cohort filtered by the proteins relevant in the models; 1: detected/positive; 0: negative; −: not detected.

**Table 3:** Best combinations differentiating infarcted and non-infarcted hearts using data from C6, C30, and C45.

C6	Uniprot	Protein Name	Generalization Capability	Accuracy	Type of Results	Secreted ^a^	Related to MI ^b^
Combination 1	P19838	NFKB1	100%	100%	Models/HT	✓	✓
	O14641	DVL2				✓	×
	Q06187	BTK				✓	×
	P01100	FOS				✓	✓
	P61981	1433G				✓	×
Combination 2	P53778	MAPK12	100%	100%	Models/HT	✓	✓
	Q9Y243	AKT3				✓	✓
	P28482	MAPK01				✓	✓
	P19838	NFKB1				✓	✓
	P45984	MAPK09				✓	✓
C30							
Combination 1	P62942	FKB1A	100%	100%	Models/HT	✓	✓
	Q14790	CASP8				✓	✓
Combination 2	P62942	FKB1A	100%	100%	Models/HT	✓	✓
	P30559	OXYR				✓	✓
C45							
Combination 1	P27487	DPP4	100%	100%	Models/HT	✓	✓
	O75330	HMMR				✓	✓
	P03952	KLKB1					
	P07203	GPX1					
	P31645	SLC6A4					
Combination 2	P62942	FKB1A	100%	100%	Models/HT	✓	✓
	P03952	KLKB1				✓	✓

^a^ Indicates whether the protein is secreted and/or has been detected in plasma. ^b^ Indicates if there is a previously known relationship between the protein and MI. Models/HT: the combinations of proteins that better classify the solution of the model to the corresponding cohort filtered by the proteins according to the high-throughput (HT) data; MI: myocardial infarction; C6: infarct core area 6 days after infarction; C30: infarct core area 30 days after infarction; C45: infarct core area 45 days after infarction.

**Table 4:** Identification of time-dependent source proteins that explain the molecular mechanism of action in myocardial infarction.

Time-Points of Protein Expression in the Core MI Area	C6	C30	C45
Proteins available for analysis ^a^	1182	1150	1033
Maximum % of proteins explained by other differential proteins ^b^	49%	48%	48%
Number of source proteins ^c^	16	18	11
% of explainable proteins explained by triggering proteins ^d^	92%	93%	89%

Proteins were identified by triggering-protein analysis. ^a^ The number of differential proteins related to cardiac remodeling available to be analyzed for each time point. ^b^ The percentage of evaluable proteins that were linked to another of the evaluated proteins (i.e., explainable by other proteins). ^c^ The number of source proteins selected. ^d^ The percentage of explainable proteins (i.e., proteins in the second row) that are explained by or linked to the selected source proteins. C: infarcted core region.

**Table 5:** Proteins involved in the common mechanism of action between time points for the infarct core region and their log ratios at each time point.

Entry Name	UniProt Code	Log Ratio C6	Log Ratio C30	Log Ratio C45
TNFA	P01375	-	-	-
RAF1	P04049	−0.69	−0.90	−0.71
P53	P04637	0.82	0.84	-
JUN	P05412	−1.49	−1.46	−1.36
IGF1R	P08069	−1.61	−0.993	−1.54
THB	P10828	−1.59	−2.30	−1.44
KPCA9	P17252	2.61	2.30	1.79
SLC9A1	P19634	-	−0.56	-
MAPK03	P27361	0.60	0.84	-
MAPK01	P28482	-	-	−0.78
MTOR	P42345	−0.57	−0.7	−0.59
TSC2	P49815	-	-	0.75
MMP14	P50281	1.32	1.74	1.40
RPS6KA3	P51812	-	-	−0.49
PTNP11	Q06124	0.86	0.58	0.64
RPS6KA2	Q15349	-	-	-
RPS6KA1	Q15418	-	-	-
BAD	Q92934	0.90	1.07	0.81

C6: infarct core area 6 days after infarction; C30: infarct core area 30 days after infarction; C45: infarct core area 45 days after infarction.

## Data Availability

Transcriptomic data is publicly available in the Gene Expression Omnibus (GEO, http://www.ncbi.nlm.nih.gov/geo/, accessed on 8 February 2019) database with series accession number GSE34569.

## Supplementary Materials

The following are available online at https://www.mdpi.com/article/10.3390/cells10123268/s1, Table S1: Initial protein filtering process to generate the truth table, Table S2: List of potential biomarkers to describe MI progression, Table S3: Enriched processes in the infarct core, Supplementary Detailed Methods: Myocardial infarction model, Tissue collection and RNA extraction, and Microarray gene expression analysis.
