# Supplementary material for: Deep Learning Analyses to Delineate the Molecular Remodeling Process after Myocardial Infarction
Source: Cells. 2021 Nov 23;10(12):3268. doi: 10.3390/cells10123268 (PMC8699769; doi:10.3390/cells10123268)
Supplement: Supplementary file 1 [file cells-10-03268-s001.zip › cells-1440494-supplementary.pdf]

cells MDPI

Supplementary Table S1. Initial protein filtering process to generate the truth table.

Localization	C6	C30	C45	R6	R30	R45
Initial entries	6047	6030	5251	127	125	21
Non- contradictory entries	5656	5697	4979	127	125	21
Single entries (no gene duplicates)	4747	4740	4212	121	116	20
Entries with a human UniProt correspondence	4737	4730	4203	121	116	20

After discarding contradictory and duplicated entries, and translating to human proteome, the number of proteins shown in row entries with a human uniprot correspondence for each cohort has been used as restrictions for the model.

Supplementary Table S2. List of potential biomarkers to describe MI progression. .

Protein Information		Identification Method	Identified as Classifier						Differential in Experimental Data				
UniProt	Protein Name		C6	C30	C45	R6	R30	R45	C30	C45	R6	R30	R45
P27487	DPP4	Models, Models/HT	1	1	1	-	1	1	-3.016	-2.022	-	-	-
Q6R327	RICTR	Models	1	1	1	-	-	1	-	-	-	-	-
Q15759	MK11	Models	1	1	1	-	-	-	-	-	-	-	-
P53778	MK12	Models, Models/HT, HT/Models	1	-	1	1	1	-	-2.185	-1.191	-	-	-
Q9Y243	AKT3	Models, Models/HT	1	-	1	-	-	-	1.417	0.959	-	-	-
P19838	NFKB1	Models/HT	1	-	1	-	-	-	-	-	-	-	-
P28482	MK01	Models/HT	1	-	1	-	-	-	-	-0.782	-	-	-
P45984	MK09	Models/HT	1	-	1	-	-	-	1.359	-	-	-	-
Q06187	BTK	Models, Models/HT	1	-	-	1	-	-	-	-	-	-	-
P01100	FOS	Models/HT	1	-	-	-	1	-	3.655	-	-	-	-
O14641	DVL2	Models, Models/HT, HT/Models	1	-	-	-	1	-	-	-	-	-	-
O75626	PRDM1	Models/HT	1	-	-	-	-	1	-	-	-	-	-
P19438	TNR1A	Models	1	-	-	-	-	-	0.86	0.674	-	-	-
Q15628	TRADD	Models	1	-	-	-	-	-	-	-	-	-	-
P19105	ML12A	Models	1	-	-	-	-	-	-	-	-	-	-
Q16539	MK14	Models	1	-	-	-	-	-	-	-	-	-	-
P61981	1433G	Models/HT	1	-	-	-	-	-	-	-	-	-	-
P02775	CXCL7	Models/HT	1	-	-	-	-	-	-	-	-	-	-
Q9UNS2	CSN3	HT	1	-	-	-	-	-	-0.506	-	-	-	-
Q9BRJ6	CG050	HT	1	-	-	-	-	-	-	-	-	-	-
P07585	PGS2	Models	-	1	1	1	-	1	2.005	1.786	-	-	-
O75676	KS6A4	Models	-	1	1	1	-	-	-	-	-	-	-
Q04724	TLE1	Models, Models/HT, HT/Models	-	1	1	-	1	-	1.968	1691	-	-	-

P62942	FKB1A	Models, Models/HT, HT/Models	-	1	1	-	-	1	-	-	-	-	-
Q6ZSZ5	ARHGI	Models	-	1	1	-	-	1	0.568	0.454	-	-	-
O75330	HMMR	Models, Models/HT	-	1	1	-	-	1	-	-	-	-	-
Q16082	HSPB2	Models, Models/HT	-	1	-	1	1	-	-	-	-	-	-
P13797	PLST	HT	-	1	-	1	-	-	1.256	0.803	-	-	-
O43541	SMAD6	Models, Models/HT	-	1	-	-	1	-	0,86	-	-	-	-
Q9NWZ3	IRAK4	Models	-	1	-	-	-	-	-	-	-	-	-
Q12778	FOXO1	Models	-	1	-	-	-	-	1.117	1.33	-	-	-
P98177	FOXO4	Models	-	1	-	-	-	-	-	-	-	-	-
P63096	GNAI1	Models	-	1	-	-	-	-	-0.683	-1.281	-	-	-
O00206	TLR4	Models	-	1	-	-	-	-	-	-	-	-	-
Q15796	SMAD2	Models	-	1	-	-	-	-	1.321	1.015	-	-	-
O60341	KDM1A	Models	-	1	-	-	-	-	-0.55	-0.505	-	-	-
Q96S42	NODAL	Models	-	1	-	-	-	-	-	-	-	-	-
Q92843	B2CL2	Models	-	1	-	-	-	-	-	-	-	-	-
Q14790	CASP8	Models/HT	-	1	-	-	-	-	-	-	-	-	-
P30559	OXYR	Models/HT	-	1	-	-	-	-	-	-	-	-	-
P06400	RB	Models/HT	-	1	-	-	-	-	-0.809	-0.521	-	-	-
P54646	AAPK2	Models/HT	-	1	-	-	-	-	-2.548	-1.092	-	-	-
Q9Y592	CEP83	HT	-	1	-	-	-	-	2.442	1.74	-	-	-
Q9UKL0	RCOR1	Models	-	-	1	1	1	-	-	-	-	-	-
Q16644	MAPK3	Models, Models/HT, HT/Models	-	-	1	1	-	1	-0.906	-0.544	-	-	-
P19525	E2AK2	Models, Models/HT	-	-	1	1	-	-	2.145	1.426	-	-	-
O15287	FANCG	Models	-	-	1	-	1	1	-	-	-	-	-
P07203	GPX1	Models, Models/HT	-	-	1	-	1	-	-	-	-	-	-
P48643	TCPE	Models, Models/HT	-	-	1	-	1	-	-	-	-	-	-
O60906	NSMA	Models/HT	-	-	1	-	1	-	-	-	-	-	-
P03952	KLKB1	Models/HT	-	-	1	-	1	-	-	-	-	-	-
P29034	S10A2	Models	-	-	1	-	-	1	-	-	-	-	-
P46013	KI67	Models	-	-	1	-	-	1	-	-	-	-	-
P21980	TGM2	Models, Models/HT	-	-	1	-	-	1	-	-	-	-	-
Q92990	GLMN	Models/HT	-	-	1	-	-	1	-	-	-	-	-
Q92974	ARHG2	Models/HT	-	-	1	-	-	1	-	-	-	-	-
Q99466	NOTC4	HT	-	-	1	-	-	1	-	-	-	-	-
Q8N1Q8	THEM5	HT	-	-	1	-	-	1	-	-	-	-	-
Q86Y07	VRK2	Models	-	-	1	-	-	-	-	-	-	-	-
P15923	TFE2	Models	-	-	1	-	-	-	-	-	-	-	-
O15264	MK13	Models	-	-	1	-	-	-	-	-	-	-	-
Q9BPZ7	SIN1	Models	-	-	1	-	-	-	-	-0.734	-	-	-
Q16099	GRIK4	Models	-	-	1	-	-	-	-	-	-	-	-
P18074	ERCC2	Models	-	-	1	-	-	-	-	-	-	-	-
P00749	UROK	Models/HT	-	-	1	-	-	-	1.384	0.871	-	-	-
Q04206	TF65	Models/HT	-	-	1	-	-	-	0.396	-	-	-	-
P31645	SC6A4	Models/HT	-	-	1	-	-	-	-	-	-	-	-

Q15797	SMAD1	Models/HT	-	-	1	-	-	-	0.657	0.552	-	-	-
P13501	CCL5	Models/HT	-	-	1	-	-	-	-	-	-	-	-
O00253	AGRP	Models/HT	-	-	1	-	-	-	-	-	-	-	-
Q9Y2C9	TLR6	Models/HT	-	-	1	-	-	-	-	-	-	-	-
P35052	GPC1	Models	-	-	-	1	1	-	-	-	-	-	-
P10966	CD8B	Models, Models/HT	-	-	-	1	-	1	-	-	-	-	-
P78347	GTF2I	Models	-	-	-	1	-	-	-0.379	-	-	-	-
P01906	DQA2	Models	-	-	-	1	-	-	-	-	-	-	-
O60239	3BP5	Models	-	-	-	1	-	-	0.702	0.852	-	-	-
O95970	LGI1	Models	-	-	-	1	-	-	-	-	-	-	-
Q13002	GRIK2	Models	-	-	-	1	-	-	-	0.664	-	-	-
P15407	FOSL1	Models	-	-	-	1	-	-	-	-	-	-	-
P17275	JUNB	Models	-	-	-	1	-	-	-	-	-	-	-
P15735	PHKG2	Models/HT	-	-	-	1	-	-	-	-	-	-	-
P01024	CO3	Models/HT	-	-	-	1	-	-	-	-	-	-	-
O00254	PAR3	Models/HT	-	-	-	1	-	-	2.55	2.161	-	-	-
P19419	ELK1	Models/HT	-	-	-	1	-	-	-	-	-	-	-
P63279	UBC9	Models/HT	-	-	-	1	-	-	0.512	-	-	-	-
Q92556	ELMO1	HT	-	-	-	1	-	-	0.936	0.669	-	-	-
Q13155	AIMP2	Models, Models/HT, HT/Models	-	-	-	-	1	1	-	-	-	-	-
P07858	CATB	Models, Models/HT	-	-	-	-	1	-	1.351	1.142	-	-	-
P12259	FA5	Models, Models/HT	-	-	-	-	1	-	-	-	-	-	-
P46940	IQGA1	Models	-	-	-	-	1	-	-	-	-	-	-
Q9BVC4	LST8	Models	-	-	-	-	1	-	-	-	-	-	-
Q9NY61	AATF	Models	-	-	-	-	1	-	-	-	-	-	-
P20339	RAB5A	Models	-	-	-	-	1	-	-	-	-	-	-
P16581	LYAM2	Models/HT	-	-	-	-	1	-	-	-	-	-	-
Q86XR7	TCAM2	Models/HT	-	-	-	-	1	-	-	-	-	-	-
P11498	PYC	Models/HT	-	-	-	-	1	-	-	-	-	-	-
Q96KB5	TOPK	Models/HT	-	-	-	-	1	-	1.238	2.053	-	-	-
Q9H1U4	MEGF9	HT	-	-	-	-	1	-	1.248	1.56	-	-	-
Q00535	CDK5	HT	-	-	-	-	1	-	-0.587	-0.417	-	-	-
Q96ST8	CEP89	Models	-	-	-	-	-	1	-	-	-	-	-
Q96NL6	SCLT1	Models	-	-	-	-	-	1	-	-	-	-	-
Q9UKV3	ACINU	Models	-	-	-	-	-	1	-	-	-	-	-
Q12913	PTPRJ	Models	-	-	-	-	-	1	2.165	1.042	-	-	-
P28222	5HT1B	Models/HT	-	-	-	-	-	1	-	-	-	-	-
P48443	RXRG	Models/HT	-	-	-	-	-	1	-2.88	-2.191	-	-	-

This table depicts the time point each protein has been detected and the log Ratio of each measurement in the microarray. Models refer to the combinations of proteins that had better classify the solutions of the models to their corresponding cohort. HT refers to the combinations of proteins that had better classify the microarray experiments to their corresponding cohort without using the information from the disease models. Models/HT refers to the combinations of proteins that had better classify the solutions of the models to their corresponding cohort, but filtering them by the proteins acting in accordance to the highthroughput (HT) data. HT/Models refers to the combinations of proteins that had better classify the microarray experiments to their corresponding cohort, filtering them by the proteins relevant in the models. 1: detected; -: Not detected.

Supplementary Table S3. Infarct Core enriched processes.

PROCESS TYPE	NAME	C6	C30	C45
GO PROCESS	VISUAL PERCEPTION	1	1	1
GO PROCESS	SKELETAL SYSTEM DEVELOPMENT	1	1	1
GO PROCESS	REGULATION OF CHONDROCYTE DIFFERENTIATION	1	1	1
GO PROCESS	RECEPTOR-MEDIATED ENDOCYTOSIS	1	1	1
GO PROCESS	PROTEIN HETEROTRIMERIZATION	1	1	1
GO PROCESS	POSITIVE REGULATION OF SYNAPSE ASSEMBLY	1	1	1
GO PROCESS	POSITIVE REGULATION OF CALCIUM ION IMPORT	1	1	1
GO FUNCTION	PLATELET-DERIVED GROWTH FACTOR BINDING	1	1	1
GO PROCESS	PLATELET DEGRANULATION	1	1	1
GO PROCESS	PLATELET ACTIVATION	1	1	1
GO PROCESS	PALATE DEVELOPMENT	1	1	1
GO PROCESS	OSSIFICATION	1	1	1
GO PROCESS	ODONTOGENESIS	1	1	1
GO PROCESS	NEGATIVE REGULATION OF ANGIOGENESIS	1	1	1
GO PROCESS	MULTICELLULAR ORGANISMAL DEVELOPMENT	1	1	1
GO PROCESS	MOVEMENT OF CELL OR SUBCELLULAR COMPONENT	1	1	1
GO PROCESS	LONG-TERM SYNAPTIC POTENTIATION	1	1	1
GO PROCESS	INTRAMEMBRANOUS OSSIFICATION	1	1	1
GO PROCESS	INTEGRIN-MEDIATED SIGNALING PATHWAY	1	1	1
GO FUNCTION	INTEGRIN BINDING	1	1	1
GO PROCESS	INNER EAR DEVELOPMENT	1	1	1
GO PROCESS	INFLAMMATORY RESPONSE	1	1	1
GO FUNCTION	IDENTICAL PROTEIN BINDING	1	1	1
GO PROCESS	HYALURONAN METABOLIC PROCESS	1	1	1
GO PROCESS	HYALURONAN CATABOLIC PROCESS	1	1	1
GO FUNCTION	HEPARIN BINDING	1	1	1
GO PROCESS	G-PROTEIN COUPLED RECEPTOR SIGNALING PATHWAY	1	1	1
GO PROCESS	GLYCOSAMINOGLYCAN METABOLIC PROCESS	1	1	1
GO FUNCTION	GLYCOSAMINOGLYCAN BINDING	1	1	1
GO PROCESS	GLOMERULAR MESANGIAL CELL DEVELOPMENT	1	1	1
GO PROCESS	FRONTAL SUTURE MORPHOGENESIS	1	1	1
GO FUNCTION	EXTRACELLULAR MATRIX STRUCTURAL CONSTITUENT	1	1	1
GO PROCESS	EXTRACELLULAR MATRIX ORGANIZATION	1	1	1
GO PROCESS	EXTRACELLULAR MATRIX DISASSEMBLY	1	1	1
GO PROCESS	EXTRACELLULAR FIBRIL ORGANIZATION	1	1	1
GO PROCESS	ENDOTHELIAL CELL-CELL ADHESION	1	1	1
GO PROCESS	ELASTIC FIBER ASSEMBLY	1	1	1
GO PROCESS	DERMATAN SULFATE BIOSYNTHETIC PROCESS	1	1	1
GO PROCESS	COLLAGEN FIBRIL ORGANIZATION	1	1	1
GO PROCESS	COLLAGEN CATABOLIC PROCESS	1	1	1
GO PROCESS	COLLAGEN BIOSYNTHETIC PROCESS	1	1	1
GO FUNCTION	COLLAGEN BINDING	1	1	1
GO PROCESS	CHONDROITIN SULFATE METABOLIC PROCESS	1	1	1
GO PROCESS	CHONDROITIN SULFATE BIOSYNTHETIC PROCESS	1	1	1
GO PROCESS	CELL ADHESION	1	1	1
GO FUNCTION	CALCIUM ION BINDING	1	1	1
GO PROCESS	BLOOD VESSEL DEVELOPMENT	1	1	1
GO PROCESS	BLOOD COAGULATION	1	1	1
GO PROCESS	AXON GUIDANCE	1	1	1
GO PROCESS	ANGIOGENESIS	1	1	1
GO PROCESS	ACTIN FILAMENT BUNDLE ASSEMBLY	1	1	1
GO FUNCTION	ACTIN FILAMENT BINDING	1	1	1
KEGG	05146_AMOEBIASIS	1	1	1
KEGG	04974_PROTEIN DIGESTION AND ABSORPTION	1	1	1

**KEGG**	04512_ECM-RECEPTOR INTERACTION	1	1	1
**KEGG**	04510_FOCAL ADHESION	1	1	1
**KEGG**	04145_PHAGOSOME	1	1	1
**KEGG**	00250_ALANINE, ASPARTATE AND GLUTAMATE METABOLISM	-1	-1	-1
**KEGG**	00260_GLYCINE, SERINE AND THREONINE METABOLISM	-1	-1	-1
**KEGG**	01100_METABOLIC PATHWAYS	-1	-1	-1
**GO FUNCTION**	2 IRON, 2 SULFUR CLUSTER BINDING	-1	-1	-1
**GO FUNCTION**	4 IRON, 4 SULFUR CLUSTER BINDING	-1	-1	-1
**GO FUNCTION**	ACYL-COA DEHYDROGENASE ACTIVITY	-1	-1	-1
**GO PROCESS**	AEROBIC RESPIRATION	-1	-1	-1
**GO PROCESS**	BRANCHED-CHAIN AMINO ACID CATABOLIC PROCESS	-1	-1	-1
**GO PROCESS**	BUNDLE OF HIS CELL-PURKINJE MYOCYTE ADHESION INVOLVED IN CELL COMMUNICATION	-1	-1	-1
**GO PROCESS**	CELLULAR METABOLIC PROCESS	-1	-1	-1
**GO PROCESS**	CELLULAR NITROGEN COMPOUND METABOLIC PROCESS	-1	-1	-1
**GO PROCESS**	CRISTAE FORMATION	-1	-1	-1
**GO FUNCTION**	ELECTRON CARRIER ACTIVITY	-1	-1	-1
**GO PROCESS**	FATTY ACID BETA-OXIDATION	-1	-1	-1
**GO PROCESS**	FATTY ACID BETA-OXIDATION USING ACYL-COA DEHYDROGENASE	-1	-1	-1
**GO FUNCTION**	FATTY-ACYL-COA BINDING	-1	-1	-1
**GO FUNCTION**	FLAVIN ADENINE DINUCLEOTIDE BINDING	-1	-1	-1
**GO PROCESS**	GLUCONEOGENESIS	-1	-1	-1
**GO PROCESS**	GLUCOSE METABOLIC PROCESS	-1	-1	-1
**GO PROCESS**	GLYCOLYTIC PROCESS	-1	-1	-1
**GO PROCESS**	LEUCINE CATABOLIC PROCESS	-1	-1	-1
**GO PROCESS**	LIPID HOMEOSTASIS	-1	-1	-1
**GO FUNCTION**	L-PHENYLALANINE	-1	-1	-1
**GO PROCESS**	MITOCHONDRIAL ATP SYNTHESIS COUPLED PROTON TRANSPORT	-1	-1	-1
**GO PROCESS**	MITOCHONDRIAL ELECTRON TRANSPORT, NADH TO UBIQUINONE	-1	-1	-1
**GO PROCESS**	MITOCHONDRIAL TRANSLATION	-1	-1	-1
**GO PROCESS**	MITOCHONDRIAL TRANSLATIONAL ELONGATION	-1	-1	-1
**GO PROCESS**	MITOCHONDRIAL TRANSLATIONAL INITIATION	-1	-1	-1
**GO PROCESS**	MITOCHONDRIAL TRANSLATIONAL TERMINATION	-1	-1	-1
**GO PROCESS**	MITOCHONDRION ORGANIZATION	-1	-1	-1
**GO FUNCTION**	NAD BINDING	-1	-1	-1
**GO FUNCTION**	NADH DEHYDROGENASE (UBIQUINONE) ACTIVITY	-1	-1	-1
**GO PROCESS**	ORGANELLE ORGANIZATION	-1	-1	-1
**GO FUNCTION**	OXIDOREDUCTASE ACTIVITY, ACTING ON THE CH-CH GROUP OF DONORS, WITH A FLAVIN AS ACCEPTOR	-1	-1	-1
**GO PROCESS**	PROTEIN HOMOTETRAMERIZATION	-1	-1	-1
**GO PROCESS**	PROTEIN TARGETING TO MITOCHONDRION	-1	-1	-1
**GO PROCESS**	PYRUVATE METABOLIC PROCESS	-1	-1	-1
**GO PROCESS**	REGULATION OF HEART RATE BY CARDIAC CONDUCTION	-1	-1	-1
**GO PROCESS**	RESPIRATORY ELECTRON TRANSPORT CHAIN	-1	-1	-1
**GO PROCESS**	SMALL MOLECULE METABOLIC PROCESS	-1	-1	-1
**GO PROCESS**	STRIATED MUSCLE CONTRACTION	-1	-1	-1
**GO FUNCTION**	STRUCTURAL CONSTITUENT OF RIBOSOME	-1	-1	-1
**GO FUNCTION**	TRANSAMINASE ACTIVITY	-1	-1	-1
**GO PROCESS**	TRICARBOXYLIC ACID CYCLE	-1	-1	-1
**GO PROCESS**	TYPE I INTERFERON SIGNALING PATHWAY	1	1	-
**GO PROCESS**	T CELL PROLIFERATION	1	1	-
**GO PROCESS**	T CELL CHEMOTAXIS	1	1	-
**GO PROCESS**	SMALL GTPASE MEDIATED SIGNAL TRANSDUCTION	1	1	-
**GO PROCESS**	RESPONSE TO VIRUS	1	1	-
**GO FUNCTION**	PROTEOGLYCAN BINDING	1	1	-
**GO PROCESS**	POSITIVE REGULATION OF MUCUS SECRETION	1	1	-
**GO PROCESS**	NEUTROPHIL CHEMOTAXIS	1	1	-

**GO PROCESS**	NEGATIVE REGULATION OF DNA REPLICATION	1	1	-
**GO FUNCTION**	LAMININ BINDING	1	1	-
**GO PROCESS**	KERATAN SULFATE BIOSYNTHETIC PROCESS	1	1	-
**GO PROCESS**	FIBRINOLYSIS	1	1	-
**GO PROCESS**	FC-GAMMA RECEPTOR SIGNALING PATHWAY INVOLVED IN PHAGOCYTOSIS	1	1	-
**GO PROCESS**	EMBRYO IMPLANTATION	1	1	-
**GO PROCESS**	DEFENSE RESPONSE TO VIRUS	1	1	-
**GO PROCESS**	CYTOKINE-MEDIATED SIGNALING PATHWAY	1	1	-
**GO PROCESS**	CELL RECOGNITION	1	1	-
**GO FUNCTION**	CALCIUM-DEPENDENT PROTEIN BINDING	1	1	-
**KEGG**	05140_LEISHMANIASIS	1	1	-
**KEGG**	04380_OSTEOCLAST DIFFERENTIATION	1	1	-
**KEGG**	00280_VALINE, LEUCINE AND ISOLEUCINE DEGRADATION	-1	-1	-
**KEGG**	00330_ARGININE AND PROLINE METABOLISM	-1	-1	-
**KEGG**	00190_OXIDATIVE PHOSPHORYLATION	-1	-1	-
**GO FUNCTION**	HYDROGEN ION TRANSMEMBRANE TRANSPORTER ACTIVITY	-1	-1	-
**GO FUNCTION**	TRANSMEMBRANE TRANSPORTER ACTIVITY	-1	-1	-
**GO PROCESS**	CELLULAR LIPID METABOLIC PROCESS	-1	-1	-
**GO PROCESS**	BIOTIN METABOLIC PROCESS	-1	-1	-
**GO PROCESS**	NADH METABOLIC PROCESS	-1	-1	-
**GO PROCESS**	MITOCHONDRIAL RESPIRATORY CHAIN COMPLEX I ASSEMBLY	-1	-1	-
**GO PROCESS**	MITOCHONDRIAL RESPIRATORY CHAIN COMPLEX III ASSEMBLY	-1	-1	-
**GO PROCESS**	MUSCLE FILAMENT SLIDING	1	-	1
**GO PROCESS**	CELL-MATRIX ADHESION	1	-	1
**GO PROCESS**	CELLULAR KETONE BODY METABOLIC PROCESS	-1	-	-1
**GO PROCESS**	GLUCOSE HOMEOSTASIS	-1	-	-1
**GO PROCESS**	POSITIVE REGULATION OF CELL DEATH	-1	-	-1
**GO PROCESS**	PROTEIN POLYUBIQUITINATION	-1	-	-1
**GO FUNCTION**	TYPE II TRANSFORMING GROWTH FACTOR BETA RECEPTOR BINDING	-	1	1
**GO FUNCTION**	RECEPTOR ACTIVITY	-	1	1
**GO PROCESS**	POSITIVE REGULATION OF CELL-SUBSTRATE ADHESION	-	1	1
**GO PROCESS**	RESPONSE TO PROGESTERONE	-	1	1
**GO PROCESS**	METANEPHROS DEVELOPMENT	-	1	1
**GO PROCESS**	POSITIVE REGULATION OF CELL DIVISION	-	1	1
**GO PROCESS**	POSITIVE REGULATION OF NEURON PROJECTION DEVELOPMENT	-	1	1
**GO PROCESS**	LOCOMOTORY BEHAVIOR	-	1	1
**GO PROCESS**	BONE MINERALIZATION	-	1	1
**GO PROCESS**	ORGAN MORPHOGENESIS	-	1	1
**GO PROCESS**	CHONDROITIN SULFATE CATABOLIC PROCESS	-	1	1
**GO PROCESS**	OVULATION CYCLE	-	1	1
**GO PROCESS**	REGULATION OF BLOOD VESSEL SIZE	-	1	1
**GO PROCESS**	POSITIVE REGULATION OF EPITHELIAL TO MESENCHYMAL TRANSITION	-	1	1
**GO PROCESS**	RESPIRATORY BURST	-	1	1
**GO PROCESS**	CARTILAGE CONDENSATION	-	1	1
**GO PROCESS**	KERATAN SULFATE METABOLIC PROCESS	-	1	1
**GO PROCESS**	NEGATIVE REGULATION OF CELL PROLIFERATION	-	1	1
**GO PROCESS**	LUNG ALVEOLUS DEVELOPMENT	-	1	1
**GO PROCESS**	AGING	-	1	1
**GO PROCESS**	NEGATIVE REGULATION OF INFLAMMATORY RESPONSE	-	1	1
**GO PROCESS**	POSITIVE REGULATION OF ASTROCYTE DIFFERENTIATION	-	1	1
**GO PROCESS**	EMBRYONIC DIGESTIVE TRACT MORPHOGENESIS	-	1	1
**GO PROCESS**	POSITIVE REGULATION OF ANGIOGENESIS	-	1	1
**GO PROCESS**	POSITIVE REGULATION OF APOPTOTIC PROCESS	-	1	1
**GO PROCESS**	BLOOD CIRCULATION	-	1	1
**GO PROCESS**	COMPLEMENT ACTIVATION, CLASSICAL PATHWAY	-	1	1
**GO PROCESS**	PHOTOTRANSDUCTION, VISIBLE LIGHT	-	1	1

**GO PROCESS**	ROUNDABOUT SIGNALING PATHWAY	-	1	1
**GO PROCESS**	ANAPHASE-PROMOTING COMPLEX-DEPENDENT PROTEASOMAL UBIQUITIN-DEPENDENT PROTEIN CATABOLIC PROCESS	-	-1	-1
**GO PROCESS**	CANONICAL GLYCOLYSIS	-	-1	-1
**GO FUNCTION**	CELL ADHESIVE PROTEIN BINDING INVOLVED IN BUNDLE OF HIS CELL-PURKINJE MYOCYTE COMMUNICATION	-	-1	-1
**GO PROCESS**	DNA DAMAGE RESPONSE, SIGNAL TRANSDUCTION BY P53 CLASS MEDIATOR RESULTING IN CELL CYCLE ARREST	-	-1	-1
**GO PROCESS**	FATTY ACID METABOLIC PROCESS	-	-1	-1
**GO PROCESS**	GLYCOGEN CATABOLIC PROCESS	-	-1	-1
**GO PROCESS**	KETONE BODY CATABOLIC PROCESS	-	-1	-1
**GO PROCESS**	POSITIVE REGULATION OF UBIQUITIN-PROTEIN LIGASE ACTIVITY INVOLVED IN REGULATION OF MITOTIC CELL CYCLE TRANSITION	-	-1	-1
**GO PROCESS**	REGULATION OF CELLULAR AMINO ACID METABOLIC PROCESS	-	-1	-1
**GO PROCESS**	REGULATION OF UBIQUITIN-PROTEIN LIGASE ACTIVITY INVOLVED IN MITOTIC CELL CYCLE	-	-1	-1
**GO PROCESS**	STARTLE RESPONSE	1	-	-
**GO FUNCTION**	SERINE-TYPE CARBOXYPEPTIDASE ACTIVITY	1	-	-
**GO PROCESS**	RESPONSE TO STIMULUS	1	-	-
**GO PROCESS**	RESPONSE TO LAMINAR FLUID SHEAR STRESS	1	-	-
**GO PROCESS**	REGULATION OF ACTIN CYTOSKELETON ORGANIZATION	1	-	-
**GO PROCESS**	PROSTATE EPITHELIAL CORD ARBORIZATION INVOLVED IN PROSTATE GLANDULAR ACINUS MORPHOGENESIS	1	-	-
**GO PROCESS**	POSITIVE REGULATION OF TUMOR NECROSIS FACTOR PRODUCTION	1	-	-
**GO PROCESS**	POSITIVE REGULATION OF INTERLEUKIN-4 PRODUCTION	1	-	-
**GO PROCESS**	POSITIVE REGULATION OF INTERLEUKIN-12 PRODUCTION	1	-	-
**GO PROCESS**	POSITIVE REGULATION OF INFLAMMATORY RESPONSE	1	-	-
**GO PROCESS**	POSITIVE REGULATION OF DENDRITIC SPINE DEVELOPMENT	1	-	-
**GO PROCESS**	POSITIVE REGULATION OF CELL MIGRATION	1	-	-
**GO PROCESS**	NEUTROPHIL ACTIVATION	1	-	-
**GO PROCESS**	NEGATIVE REGULATION OF INTERLEUKIN-17 PRODUCTION	1	-	-
**GO PROCESS**	MONOCYTE CHEMOTAXIS	1	-	-
**GO PROCESS**	MACROPHAGE CHEMOTAXIS	1	-	-
**GO FUNCTION**	LIPOPOLYSACCHARIDE RECEPTOR ACTIVITY	1	-	-
**GO FUNCTION**	HYALURONIC ACID BINDING	1	-	-
**GO PROCESS**	HINDBRAIN DEVELOPMENT	1	-	-
**GO FUNCTION**	GTPASE ACTIVATOR ACTIVITY	1	-	-
**GO PROCESS**	DEVELOPMENTAL GROWTH	1	-	-
**GO PROCESS**	DENDRITIC CELL CHEMOTAXIS	1	-	-
**GO PROCESS**	CYTOKINE PRODUCTION	1	-	-
**GO PROCESS**	CHRONIC INFLAMMATORY RESPONSE	1	-	-
**GO PROCESS**	CHEMOTAXIS	1	-	-
**GO PROCESS**	CHEMOKINE-MEDIATED SIGNALING PATHWAY	1	-	-
**GO FUNCTION**	CHEMOKINE ACTIVITY	1	-	-
**GO PROCESS**	CELLULAR RESPONSE TO FIBROBLAST GROWTH FACTOR STIMULUS	1	-	-
**GO FUNCTION**	ACTIN MONOMER BINDING	1	-	-
**GO FUNCTION**	ACTIN BINDING	1	-	-
**KEGG**	00071_FATTY ACID DEGRADATION	-1	-	-
**KEGG**	04270_VASCULAR SMOOTH MUSCLE CONTRACTION	-1	-	-
**GO FUNCTION**	LBD DOMAIN BINDING	-1	-	-
**GO FUNCTION**	TITIN BINDING	-1	-	-
**GO FUNCTION**	BIOTIN CARBOXYLASE ACTIVITY	-1	-	-
**GO FUNCTION**	CYSTEINE-TYPE ENDOPEPTIDASE INHIBITOR ACTIVITY INVOLVED IN APOPTOTIC PROCESS	-1	-	-
**GO FUNCTION**	OXIDOREDUCTASE ACTIVITY	-1	-	-
**GO FUNCTION**	INTERLEUKIN-1 RECEPTOR ACTIVITY	-1	-	-
**GO PROCESS**	CELLULAR RESPONSE TO DRUG	-1	-	-

**GO PROCESS**	POSITIVE REGULATION OF VASCULAR PERMEABILITY	-1	-	-
**GO PROCESS**	POSITIVE REGULATION OF LEUKOCYTE MIGRATION	-1	-	-
**GO PROCESS**	KETONE BODY BIOSYNTHETIC PROCESS	-1	-	-
**GO PROCESS**	POSITIVE REGULATION OF GLUCOSE METABOLIC PROCESS	-1	-	-
**GO PROCESS**	BEHAVIORAL RESPONSE TO COCAINE	-1	-	-
**GO PROCESS**	MEMBRANE DEPOLARIZATION DURING ACTION POTENTIAL	-1	-	-
**GO PROCESS**	REGULATION OF PROTEASOMAL UBIQUITIN-DEPENDENT PROTEIN CATABOLIC PROCESS	-1	-	-
**GO PROCESS**	POSITIVE REGULATION OF VASCULAR ENDOTHELIAL GROWTH FACTOR RECEPTOR SIGNALING PATHWAY	-1	-	-
**GO PROCESS**	NEGATIVE REGULATION OF OXIDATIVE STRESS-INDUCED INTRINSIC APOPTOTIC SIGNALING PATHWAY	-1	-	-
**GO PROCESS**	LABYRINTHINE LAYER DEVELOPMENT	-1	-	-
**GO PROCESS**	ASPARTATE METABOLIC PROCESS	-1	-	-
**GO PROCESS**	POSITIVE REGULATION OF SODIUM ION TRANSPORT	-1	-	-
**GO PROCESS**	REGULATION OF SMOOTH MUSCLE CELL DIFFERENTIATION	-1	-	-
**GO PROCESS**	RESPONSE TO MUSCLE STRETCH	-1	-	-
**GO PROCESS**	POSITIVE REGULATION OF PROTEIN KINASE ACTIVITY	-1	-	-
**GO PROCESS**	EMBRYONIC HEART TUBE LEFT/RIGHT PATTERN FORMATION	-1	-	-
**GO PROCESS**	MALE SEX DETERMINATION	-1	-	-
**GO PROCESS**	POTASSIUM ION EXPORT	-1	-	-
**KEGG**	04620_TOLL-LIKE RECEPTOR SIGNALING PATHWAY	-	1	-
**GO FUNCTION**	CALCIUM-DEPENDENT PHOSPHOLIPID BINDING	-	1	-
**GO FUNCTION**	SERINE-TYPE ENDOPEPTIDASE INHIBITOR ACTIVITY	-	1	-
**GO FUNCTION**	PHOSPHATIDYLSERINE BINDING	-	1	-
**GO FUNCTION**	TRANSFORMING GROWTH FACTOR BETA BINDING	-	1	-
**GO FUNCTION**	TYPE III TRANSFORMING GROWTH FACTOR BETA RECEPTOR BINDING	-	1	-
**GO FUNCTION**	PEPTIDASE ACTIVATOR ACTIVITY	-	1	-
**GO PROCESS**	BLOOD VESSEL REMODELING	-	1	-
**GO PROCESS**	POSITIVE REGULATION OF ERYTHROCYTE DIFFERENTIATION	-	1	-
**GO PROCESS**	CELL PROLIFERATION	-	1	-
**GO PROCESS**	SRP-DEPENDENT COTRANSLATIONAL PROTEIN TARGETING TO MEMBRANE	-	1	-
**GO PROCESS**	RESPONSE TO WOUNDING	-	1	-
**GO PROCESS**	HIGH-DENSITY LIPOPROTEIN PARTICLE ASSEMBLY	-	1	-
**GO PROCESS**	RETINOID METABOLIC PROCESS	-	1	-
**GO PROCESS**	REGULATION OF CELL MIGRATION	-	1	-
**GO PROCESS**	OLIGODENDROCYTE DIFFERENTIATION	-	1	-
**GO PROCESS**	NEGATIVE REGULATION OF NEURON DIFFERENTIATION	-	1	-
**GO PROCESS**	AXON EXTENSION INVOLVED IN AXON GUIDANCE	-	1	-
**GO PROCESS**	NOTCH SIGNALING PATHWAY	-	1	-
**GO PROCESS**	RESPONSE TO ESTRADIOL	-	1	-
**GO PROCESS**	NEGATIVE REGULATION OF MAP KINASE ACTIVITY	-	1	-
**GO PROCESS**	RETINA DEVELOPMENT IN CAMERA-TYPE EYE	-	1	-
**GO PROCESS**	KERATAN SULFATE CATABOLIC PROCESS	-	1	-
**GO PROCESS**	RESPONSE TO PEPTIDE HORMONE	-	1	-
**GO PROCESS**	NEUROPEPTIDE SIGNALING PATHWAY	-	1	-
**GO PROCESS**	NEGATIVE REGULATION OF CARTILAGE DEVELOPMENT	-	1	-
**GO PROCESS**	BEHAVIORAL RESPONSE TO PAIN	-	1	-
**GO PROCESS**	NEGATIVE REGULATION OF PLASMINOGEN ACTIVATION	-	1	-
**GO PROCESS**	POSITIVE REGULATION OF CHOLESTEROL ESTERIFICATION	-	1	-
**GO PROCESS**	POSITIVE REGULATION OF LIPOPOLYSACCHARIDE-MEDIATED SIGNALING PATHWAY	-	1	-
**GO PROCESS**	BLEB ASSEMBLY	-	1	-
**GO PROCESS**	OSTEOBLAST DIFFERENTIATION	-	1	-
**GO PROCESS**	INTERFERON-GAMMA-MEDIATED SIGNALING PATHWAY	-	1	-
**GO PROCESS**	SOMATIC STEM CELL MAINTENANCE	-	1	-

**GO PROCESS**	POSITIVE REGULATION OF INTERLEUKIN-6 PRODUCTION	-	1	-
**GO PROCESS**	POSITIVE REGULATION OF BMP SIGNALING PATHWAY	-	1	-
**GO PROCESS**	INNATE IMMUNE RESPONSE	-	1	-
**GO PROCESS**	CYCLOOXYGENASE PATHWAY	-	1	-
**GO PROCESS**	PHOSPHOLIPID EFFLUX	-	1	-
**GO PROCESS**	REGULATION OF CELL GROWTH	-	1	-
**GO PROCESS**	POSITIVE REGULATION OF FAT CELL DIFFERENTIATION	-	1	-
**GO PROCESS**	RESPONSE TO NUTRIENT LEVELS	-	1	-
**GO PROCESS**	CELLULAR DEFENSE RESPONSE	-	1	-
**GO PROCESS**	POSITIVE REGULATION OF CELL ADHESION MEDIATED BY INTEGRIN	-	1	-
**GO PROCESS**	REGULATION OF ATPASE ACTIVITY	-	1	-
**KEGG**	00910_NITROGEN METABOLISM	-	-1	-
**KEGG**	00270_CYSTEINE AND METHIONINE METABOLISM	-	-1	-
**GO FUNCTION**	GLYCOGEN PHOSPHORYLASE ACTIVITY	-	-1	-
**GO FUNCTION**	PYRIDOXAL PHOSPHATE BINDING	-	-1	-
**GO PROCESS**	VENTRICULAR CARDIAC MUSCLE CELL ACTION POTENTIAL	-	-1	-
**GO PROCESS**	HYDROGEN ION TRANSMEMBRANE TRANSPORT	-	-1	-
**GO PROCESS**	CELLULAR AMINO ACID BIOSYNTHETIC PROCESS	-	-1	-
**GO PROCESS**	OXIDATIVE PHOSPHORYLATION	-	-1	-
**GO PROCESS**	MITOCHONDRIAL ELECTRON TRANSPORT, CYTOCHROME C TO OXYGEN	-	-1	-
**GO PROCESS**	RESPONSE TO MUSCLE ACTIVITY	-	-1	-
**GO PROCESS**	SMOOTH MUSCLE CELL DIFFERENTIATION	-	-1	-
**GO PROCESS**	MEMBRANE REPOLARIZATION DURING ACTION POTENTIAL	-	-1	-
**GO PROCESS**	REGULATION OF VENTRICULAR CARDIAC MUSCLE CELL ACTION POTENTIAL	-	-1	-
**GO PROCESS**	EMBRYONIC AXIS SPECIFICATION	-	-1	-
**GO PROCESS**	NEGATIVE REGULATION OF GLUCOCORTICOID RECEPTOR SIGNALING PATHWAY	-	-1	-
**GO PROCESS**	MITOCHONDRION MORPHOGENESIS	-	-1	-
**GO PROCESS**	ATP BIOSYNTHETIC PROCESS	-	-1	-
**GO PROCESS**	POSITIVE REGULATION OF HEART RATE	-	-1	-
**GO PROCESS**	SUCCINATE METABOLIC PROCESS	-	-1	-
**GO PROCESS**	L-PHENYLALANINE CATABOLIC PROCESS	-	-1	-
**GO PROCESS**	REGULATION OF ACETYL-COA BIOSYNTHETIC PROCESS FROM PYRUVATE	-	-1	-
**GO PROCESS**	REGULATION OF POTASSIUM ION TRANSMEMBRANE TRANSPORT	-	-1	-
**KEGG**	05110_VIBRIO CHOLERAE INFECTION	-	-	1
**GO FUNCTION**	TRANSFERASE ACTIVITY, TRANSFERRING GLYCOSYL GROUPS	-	-	1
**GO FUNCTION**	TROPOMYOSIN BINDING	-	-	1
**GO FUNCTION**	ROUNDABOUT BINDING	-	-	1
**GO FUNCTION**	EXTRACELLULAR MATRIX CONSTITUENT CONFERRING ELASTICITY	-	-	1
**GO FUNCTION**	PROTEIN COMPLEX BINDING	-	-	1
**GO PROCESS**	CAMERA-TYPE EYE DEVELOPMENT	-	-	1
**GO PROCESS**	SENSORY PERCEPTION OF PAIN	-	-	1
**GO PROCESS**	EMBRYONIC EYE MORPHOGENESIS	-	-	1
**GO PROCESS**	CELL MOTILITY	-	-	1
**GO PROCESS**	EXTRINSIC APOPTOTIC SIGNALING PATHWAY	-	-	1
**GO PROCESS**	GLIAL CELL MIGRATION	-	-	1
**GO PROCESS**	BONE TRABECULA FORMATION	-	-	1
**GO PROCESS**	NEGATIVE REGULATION OF CHEMOKINE-MEDIATED SIGNALING PATHWAY	-	-	1
**GO PROCESS**	CELL GROWTH	-	-	1
**GO PROCESS**	ENDOTHELIAL CELL PROLIFERATION	-	-	1
**GO PROCESS**	CARBOHYDRATE METABOLIC PROCESS	-	-	1
**GO PROCESS**	POSITIVE REGULATION OF EXECUTION PHASE OF APOPTOSIS	-	-	1
**GO PROCESS**	BONE RESORPTION	-	-	1

**GO PROCESS**	TRANSITION BETWEEN FAST AND SLOW FIBER	-	-	1
**GO PROCESS**	GLYCOSAMINOGLYCAN BIOSYNTHETIC PROCESS	-	-	1
**GO PROCESS**	SUBSTRATE ADHESION-DEPENDENT CELL SPREADING	-	-	1
**GO PROCESS**	POSITIVE REGULATION OF RAS PROTEIN SIGNAL TRANSDUCTION	-	-	1
**GO PROCESS**	TISSUE REMODELING	-	-	1
**GO PROCESS**	MUSCLE ORGAN DEVELOPMENT	-	-	1
**GO PROCESS**	REGULATION OF CELLULAR RESPONSE TO GROWTH FACTOR STIMULUS	-	-	1
**GO PROCESS**	POSITIVE REGULATION OF CHONDROCYTE DIFFERENTIATION	-	-	1
**GO PROCESS**	CELL JUNCTION ASSEMBLY	-	-	1
**GO PROCESS**	CELLULAR RESPONSE TO RETINOIC ACID	-	-	1
**GO PROCESS**	CELLULAR RESPONSE TO AMINO ACID STIMULUS	-	-	1
**GO PROCESS**	POSITIVE REGULATION OF CHOLESTEROL EFFLUX	-	-	1
**GO PROCESS**	3-PHOSPHOADENOSINE 5-PHOSPHOSULFATE METABOLIC PROCESS	-	-	1
**GO PROCESS**	HEPARAN SULFATE PROTEOGLYCAN BIOSYNTHETIC PROCESS	-	-	1
**GO PROCESS**	CELLULAR GLUCOSE HOMEOSTASIS	-	-	1
**GO PROCESS**	POSITIVE REGULATION OF CATENIN IMPORT INTO NUCLEUS	-	-	1
**GO PROCESS**	MUSCLE CONTRACTION	-	-	1
**GO PROCESS**	BODY FLUID SECRETION	-	-	1
**GO PROCESS**	ADENYLATE CYCLASE-ACTIVATING G-PROTEIN COUPLED RECEPTOR SIGNALING PATHWAY	-	-	1
**KEGG**	00100_STEROID BIOSYNTHESIS	-	-	-1
**GO PROCESS**	GLYCOGEN METABOLIC PROCESS	-	-	-1
**GO PROCESS**	HEME BIOSYNTHETIC PROCESS	-	-	-1
**GO PROCESS**	MEMBRANE REPOLARIZATION DURING CARDIAC MUSCLE CELL ACTION POTENTIAL	-	-	-1
**GO PROCESS**	NEGATIVE REGULATION OF NOTCH SIGNALING PATHWAY	-	-	-1
**GO PROCESS**	NEGATIVE REGULATION OF UBIQUITIN-PROTEIN LIGASE ACTIVITY INVOLVED IN MITOTIC CELL CYCLE	-	-	-1
**GO PROCESS**	NOREPINEPHRINE METABOLIC PROCESS	-	-	-1
**GO PROCESS**	OXALOACETATE METABOLIC PROCESS	-	-	-1
**GO FUNCTION**	PHOSPHATIDYLINOSITOL 3-KINASE BINDING	-	-	-1
**GO FUNCTION**	PHOSPHOLIPASE ACTIVITY	-	-	-1
**GO PROCESS**	REGULATION OF TRANSLATIONAL TERMINATION	-	-	-1
**GO PROCESS**	RELEASE OF CYTOCHROME C FROM MITOCHONDRIA	-	-	-1
**GO PROCESS**	SHORT-CHAIN FATTY ACID CATABOLIC PROCESS	-	-	-1
**GO PROCESS**	SIALYLATION	-	-	-1
**GO FUNCTION**	SIGNALING ADAPTOR ACTIVITY	-	-	-1
**GO PROCESS**	STIMULATORY C-TYPE LECTIN RECEPTOR SIGNALING PATHWAY	-	-	-1
**GO PROCESS**	VENTRICULAR SEPTUM MORPHOGENESIS	-	-	-1
